# Long-read 16S rRNA amplicon sequencing reveals microbial characteristics in patients with colorectal adenomas and carcinoma lesions in Egypt

**DOI:** 10.1186/s13099-025-00681-9

**Published:** 2025-02-02

**Authors:** Asmaa A. El Leithy, Amira Salah El-Din Youssef, Auhood Nassar, Ramy K. Aziz, Nadin M. Khaled, Mina T. Mahrous, Ghobrial N. Farahat, Aya H. Mohamed, Yasser Mabrouk Bakr

**Affiliations:** 1 College of Biotechnology, Misr University for Science and Technology, Giza, Egypt; 2 Virology and Immunology Unit, Cancer Biology Department, National Cancer Institute, Cairo University, Kasr Al-Aini st., Fom El-Khaleeg, Cairo, 11976 Egypt; 3 Department of Microbiology and Immunology, Faculty of Pharmacy, Cairo University, Cairo, Egypt; 4 Center for Genome and Microbiome Research, Cairo University, Cairo, Egypt; 5 Cancer Biology Department, National Cancer Institute, Cairo University, Cairo, Egypt

**Keywords:** Microbiome, 16S rRNA, Colorectal cancer, Colonic polyps

## Abstract

**Background:**

Colorectal cancer (CRC) is among the five leading causes of cancer incidence and mortality. During the past decade, the role of the gut microbiota and its dysbiosis in colorectal tumorigenesis has been emphasized. Metagenomics and amplicon-based microbiome profiling provided insights into the potential role of microbial dysbiosis in the development of CRC.

**Aim:**

To address the scarcity of information on differential microbiome composition of tumor tissue in comparison to adenomas and the lack of such data from Egyptian patients with CRC.

**Materials and methods:**

Long-read nanopore sequencing of 16S rRNA amplicons was used to profile the colonic microbiota from fresh colonoscopic biopsy samples of Egyptian patients with CRC and patients with colonic polyps.

**Results:**

Species richness of CRC lesions was significantly higher than that in colonic polyps (*p*-value = 0.0078), while evenness of the CRC group was significantly lower than the colonic polyps group (*p*-value = 0.0055). Both species richness and Shannon diversity index of the late onset CRC samples were significantly higher than those of the early onset ones. The Firmicutes-to-Bacteroidetes (F/B) ratio was significantly higher in the CRC group than in the colonic polyps group (*p*-value = 0.0054), and significantly higher in samples from early-onset CRC. The *Enterococcus* spp. were significantly overabundant in patients with rectal cancer and early-onset CRC, while *Staphylococcus* spp. were significantly higher in patients with sigmoid cancer and late-onset CRC. In addition, the relative abundance of *Fusobacterium nucleatum* was significantly higher in CRC patients.

**Conclusion:**

Differentiating trends were identified at phylum, genus, and species levels, despite the inter-individual differences. In summary, this study addressed the microbial dysbiosis associated with CRC and colonic polyps groups, paving the way for a better understanding of the pathogenesis of early and late-onset CRC in Egyptian patients.

**Supplementary Information:**

The online version contains supplementary material available at 10.1186/s13099-025-00681-9.

## Introduction

Colorectal cancer (CRC) is the third most frequently diagnosed cancer, in terms of incidence, and the second leading cause of cancer mortality (9.3% of the total cancer deaths). In men, it is the third cancer site with the highest age-standardized rate in countries with higher human development index [1].

CRC mostly arises from colorectal polyps (adenoma) as the polyps are prone to malignant carcinoma transformation [2]. Genetic mutations associated with the disease progression in Egyptian patients with CRC have been recently described [3]. CRC development is multifactorial, with a strong genetic component. However, it is also one of the most lifestyle-affected cancers, since the colon is directly connected to diet and various dietary pollutants. Additionally, the past decade emphasized the role of the gut microbiota and its dysbiosis in colorectal tumorigenesis, which might be a causative change [4].

Recent advancements in high-throughput sequencing technologies, such as shotgun metagenomics and 16S rRNA amplicon sequencing, have notably improved the understanding of the role of microbiome in CRC progression [5]. Fortunately, in the past few years, long-read sequencing, such as the nanopore technology, has improved in accuracy and dropped in price. It offers many advantages over the most widely used short-read sequencing approaches, most importantly the ability to resolve differences between species with near-identical rRNA variable regions, since nanopores allow the sequencing of the full 16S rRNA gene, eliminating phylogenetic biases [6].

Metagenomics and amplicon-based microbiome profiling provided insights into the potential role of microbial dysbiosis in the development of CRC. Dozens of studies delineated specific bacterial taxa and CRC-associated functional pathways [7]. In addition, biomarkers for early detection and prevention of CRC are also being identified. For example, a panel of 16 bacterial markers could differentiate between CRC patients and healthy controls with 92% accuracy [8].

Bacterial phyla, such as Firmicutes and Bacteroidetes, were pinpointed as underrepresented in patients with cancer, compared with healthy individuals. However, *Prevotella copri, Mansonia uniformis, Fusobacterium nucleatum*, and specific strains of *Escherichia coli* have been described as overabundant in cancer groups [9].

In addition to the altered microbiota makeup, pathogenic bacterial species might contribute to the emergence of CRC such as several *Bacteroides* species (*B. vulgatus,B. fragilis*, and *B. stercoris*), *Bifidobacterium angulatum*, some *Ruminococcus* species, *Fusobacterium prausnitzii* [10]. Such microbes are believed to induce CRC tumorigenesis by promoting the proliferation of the epithelial cells, producing epithelial barrier damage and causing inflammation. Moreover, different toxins may damage DNA, stimulating the pro-tumorigenic effect. For example, *Bacteroides fragilis* toxin is reported to activate Wnt and NF-kB signaling pathways and induce the epithelial release of pro-inflammatory molecules [11].

A growing body of evidence supports that the microbiome can influence response to immunotherapy and chemotherapy [12, 13]. Modulating the microbiome may provide methods to increase the efficacy of treatments, reduce treatment toxicities, and even prevent carcinogenesis. While research on the fecal microbiome has been frequently conducted, little is known about the role of tissue microbiota in determining disease associations and the diagnostic and therapeutic potential of the microbiome in Egyptian patients with CRC [14].

In Egypt, only a handful studies have addressed the microbiome involvement in CRC, yet these studies were based on fecal microbiome profiling, or fecal analysis by real-time PCR [15–19] but none profiled the tumor tissue.

Thus, this study was launched to address the scarcity of information about the tissue microbiome composition by using long-read sequencing to compare the microbiomes of CRC tumors and polyps, specifically tackle the lack of any such data from Egyptian patients with CRC, given the importance of geographical and dietary factors shaping the microbiome. In addition, we identified microbiome variations associated with early and late-onset CRC, as well as anatomical tumor site.

## Materials and methods

### Ethics statement

All protocols and procedures were approved by the Institutional Review Board (IRB approval number: CB2309-302-071) of National Cancer Institute (NCI), Cairo University, Egypt. Written informed consent was obtained from each participant before their enrolment in this study.

### Sample collection and description

Fresh colonoscopic biopsy samples (n = 15) from CRC patients and patients with colonic polyps (n = 14) were recruited from the NCI of Egypt. The collected biopsies were stored in MACS Tissue Storage Solution in a − 80 freezer until DNA extraction. All the participants’ clinicopathological data were collected from their National Cancer Institute (NCI) clinical records.

### DNA extraction

DNA was isolated from the collected biopsies using the QIAamp^®^ DNA mini kit (Cat. No. 51304, Qiagen, Germany) following the manufacturer’s instructions. The concentration of the purified DNA was measured using Qubit^®^ 3.0 Fluorometer (Cat. No, Q33216, Thermo Fischer Scientific Inc., USA) with Qubit™ dsDNA BR assay kit (Cat. No. Q32850, Thermo Fischer Scientific Inc., USA).

### Amplification of 16S rRNA

PCR amplification for the 16S rRNA gene was performed using the 27 F/1492R primer set from the 16S Barcoding Kit (SQK-RAB204; Nanopore Technologies, Oxford, UK) and PCRBIO HS Taq Mix Red (PCR Biosystems Ltd., London, Oxford, UK) according to the manufacturer’s protocol in a reaction volume of 50 µL consisting of 10 ng of genomic DNA and 1 µl 16S barcoded primers at 10 µM. The thermal profile for the amplification was as follows: initial denaturation at 95 °C for 2 min, 30 cycles of 95 °C for 20 s, 55 °C for 30 s, and 72 °C for 45 s, followed by a final extension at 72 °C for 5 min, using MyCycler Thermal Cycler (Bio-Rad, California, USA) following the manufacturer’s instructions.

### Library preparation and sequencing for the 16S rRNA amplified PCR products

The PCR amplicons were pooled then purified using AMPure XP (Beckman Coulter, Indianapolis, IN, USA) and quantified using Qubit 4 (Thermo Fischer Scientific). A total of 200 ng DNA was used for library preparation; the resulting library was sequenced with MinIon™ sequencer (Nanopore Technologies, Oxford, UK) for almost 24 h using R9.4.1 flow cells (FLO-MIN106; Oxford Nanopore Technologies) according to the manufacturer’s instructions. Minknow software version 22.12.7 (Oxford Nanopore Technologies) was used for real-time base-calling for sequenced data.

### Bioinformatics and statistical analysis

The Guppy software V6.4.6 (Oxford Nanopore Technologies) was used for base-calling, adapter/barcode trimming, and then to generate FASTQ-formatted sequence files, with an accurate base-calling model and read filtering of min_ score = 9 and reads below Q9 were eliminated. The FASTQ reads were assigned to their taxonomic group by alignment to the NCBI 16S database.

After relative taxon abundance data were obtained, all data were analyzed for statistical significance and visualized by publicly available packages in the R environment (https://www.r-project.org/) and the RStudio software (version 2022.02.0 Build 443). The following R packages were used: *readxl, dplyr, tidyverse, ggplot2, ggpubr, corrplot, magrittr, pheatmap*, and *coin*.

All comparisons between two variables were tested for statistical significance by the non-parametric Mann-Whitney test, while comparisons between multiple variables were tested for significance by the Kruskal-Wallis test, followed by *post hoc* Mann-Whitney tests with Tucky adjustment. Correlation analysis was performed by the Spearman non-parametric method.

## Results

### Patients’ clinical data and metadata

Age, sex, histological type, site, grade, and state of metastasis or recurrence for all participants are summarized in Table 1.

**Table 1:** Demographic and clinical data of the studied patients

	CRC *n* = 15	Colonic polyps *n* = 14
Age: Early age ≤ 45 Late age > 45	4 (27%) 11 (73%)	3 (21%) 11 (79%)
Sex Male Female	5 (33%) 10 (67%)	6 (43%) 8 (47%)
Histological type	Adenocarcinoma 12 (80%) Mucinous adenocarcinoma 1 (7%) Signet Ring adenocarcinoma 2 (13%)	Typical lesion 5 (34%) Atypical lesion 6 (45%) Mixed lesions 3 (21%)
Site Colon Rectum Sigmoid	7 (47%) 6 (40%) 2 (13%)	13 (93%) 0 (0%) 1 (7%)
Grade II NA	13 (87%) 2 (13%)	-
Metastasis Yes No	0 (0%) 15 (100%)	-
Recurrence Yes No	5 (33%) 10 (67%)	-

### Microbial composition of the studied patients with CRC and colonic polyps

**Fig. 1:**
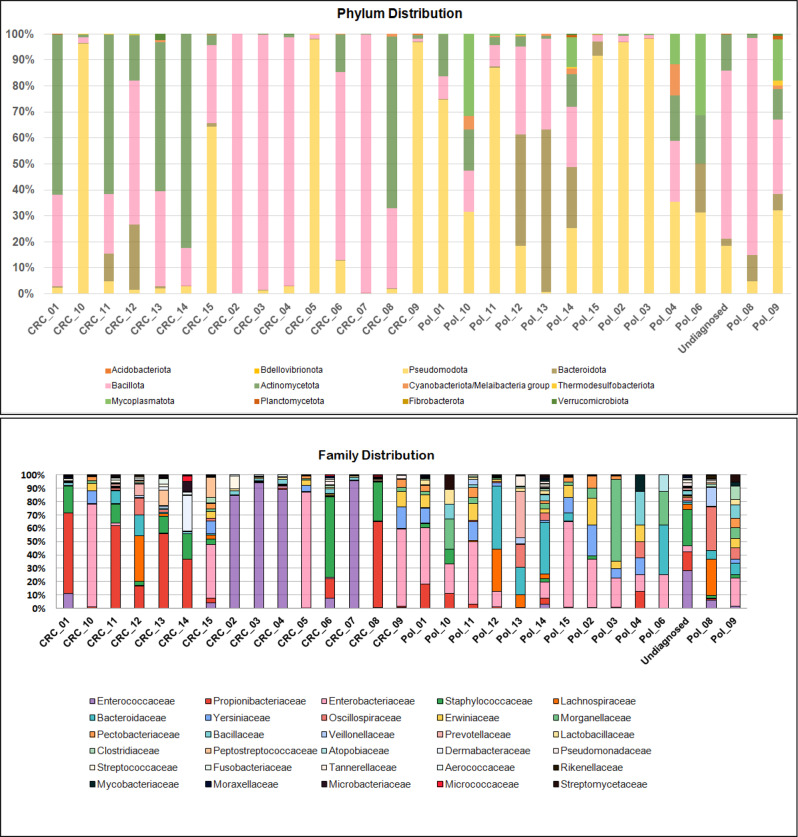
Distribution of bacterial phyla and families in CRC and colonic polyps groups. Y axis: relative abundance (% of total reads) of reads assigned to the indicated phyla, after unassigned sequences were excluded. X axis: different samples from patients with CRC, polyps, and one ambiguous, undiagnosed case

Nanopore sequences of the full 16S rRNA gene from all the study participants were generated and filtered for quality, then assigned to different taxonomic levels. Despite obvious inter-individual differences, differentiating trends could still be identified. At the phylum level, Firmicutes and Actinomycetota were overabundant in the CRC group. At the family level, several samples from the CRC group were highly enriched in family *Enterococcaceae*, while *Bacteroidaceae* was more represented in the colonic polyps (Fig. 1). The significantly abundant bacterial phyla and families in CRC and colonic polyps are presented in Supplementary Table 2.

**Fig. 2:**
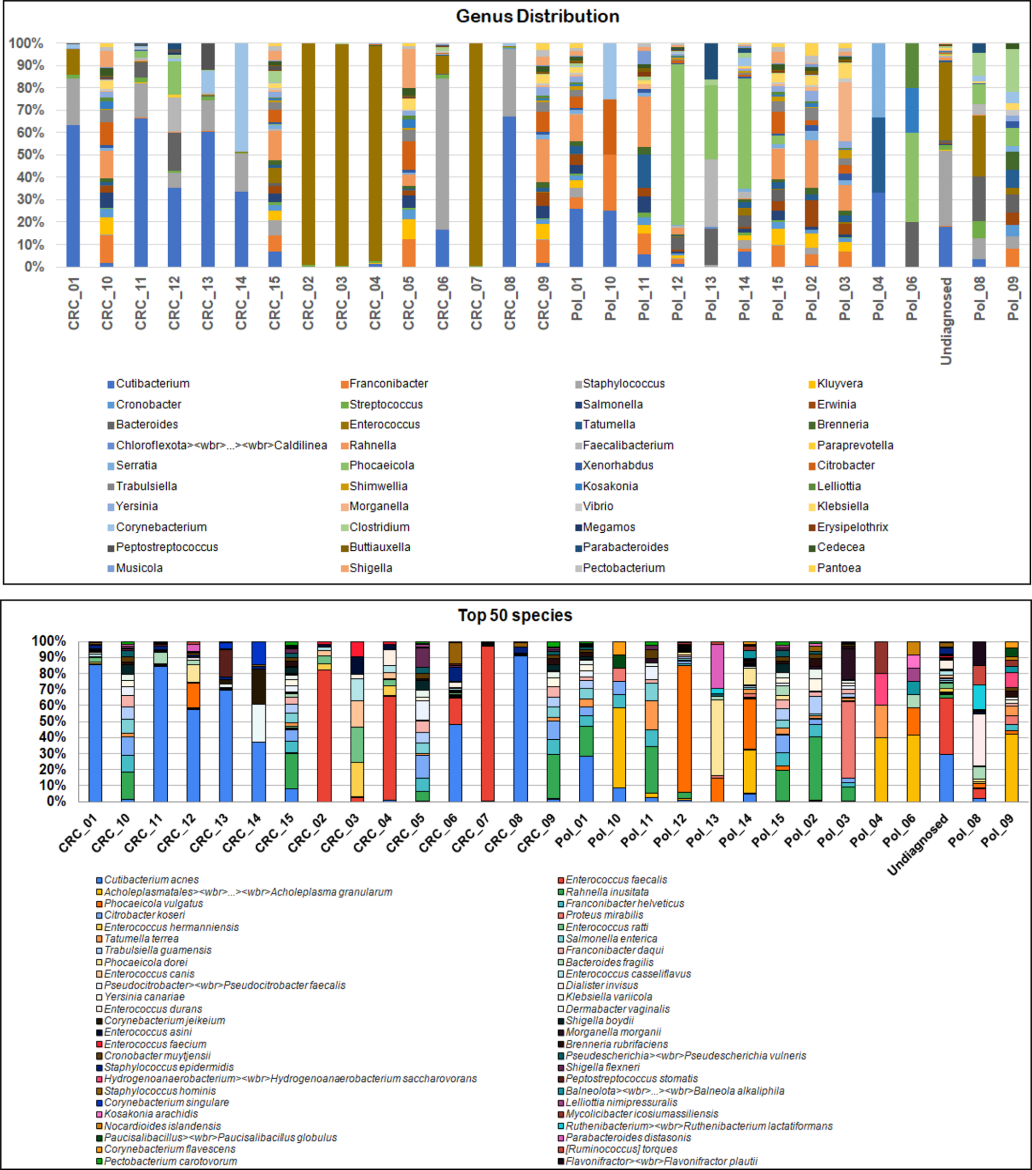
Genus distribution (Top panel) and the top 50 species (bottom panel) of the current studied samples

At the genus level, multiple genera were significantly associated with CRC tissue, such as *Enterococcus,Cutibacterium,Staphylococcus,Corynebacterium,Peptostreptococcus*, and *Fusobacterium*. On the other hand, genera associated with colonic polyps included *Proteus,Prevotella,Bacteroides*, and *Macrococcus* (Fig. 2). Abundant bacterial genera and species among CRC and colonic polyps are presented in Supplementary Table 3.

### Signature microbiome profiles in patients with CRC and colonic polyps

**Fig. 3:**
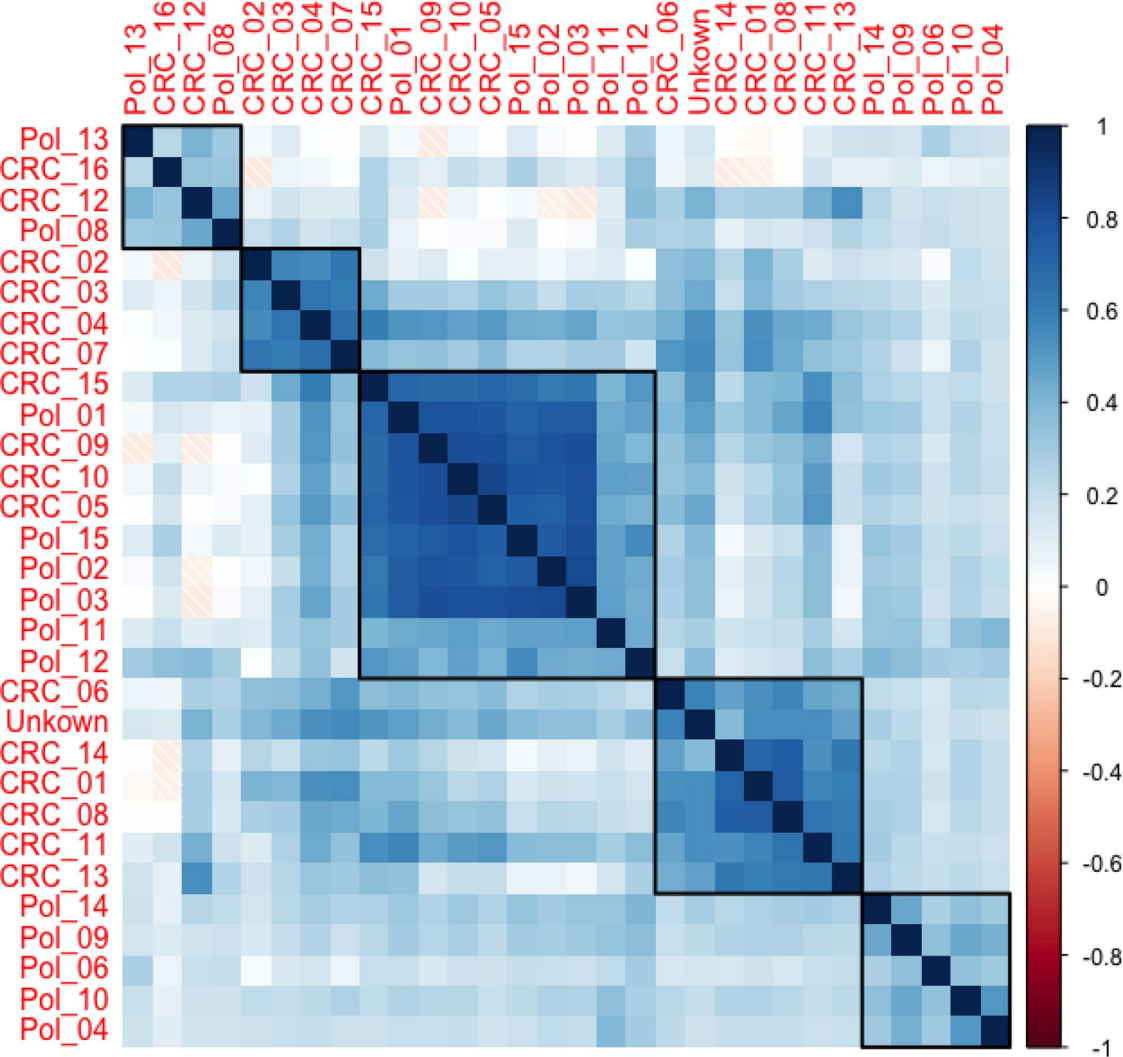
A color-coded correlation plot indicates five clusters of correlated relative abundance within the microbiome profiles of samples from CRC tissue and colonic polyps

Using correlation analysis of taxon abundance profiles within different samples delineated five clusters, three of which are clearly associated with the type of lesion (Clusters B, D, and E in Fig. 3). Similar clustering analysis (Supplementary Fig. 1) identified two clear clusters in tissues of the CRC groups. In Cluster 1, *Cutibacterium* (17.01%), *Staphylococcus* (8.60%), *Corynebacterium* (2.86%), *Dermabacter* (1.41%), and *Peptostreptococcus* (0.91%) significantly dominated the tissue microbiota of CRC patients (samples CRC_08, CRC_11, CRC_12, CRC_13, CRC_14, four of which are members of Correlation Cluster D in Fig. 3). Cluster 2 was characterized by a significant overabundance of *Enterococcus* (24.28%) in the tissue of CRC patients (samples CRC_02, CRC_03, CRC_04, CRC_07, which are all members of Correlation Cluster B in Fig. 3).

### Alpha diversity variations among samples

**Fig. 4:**
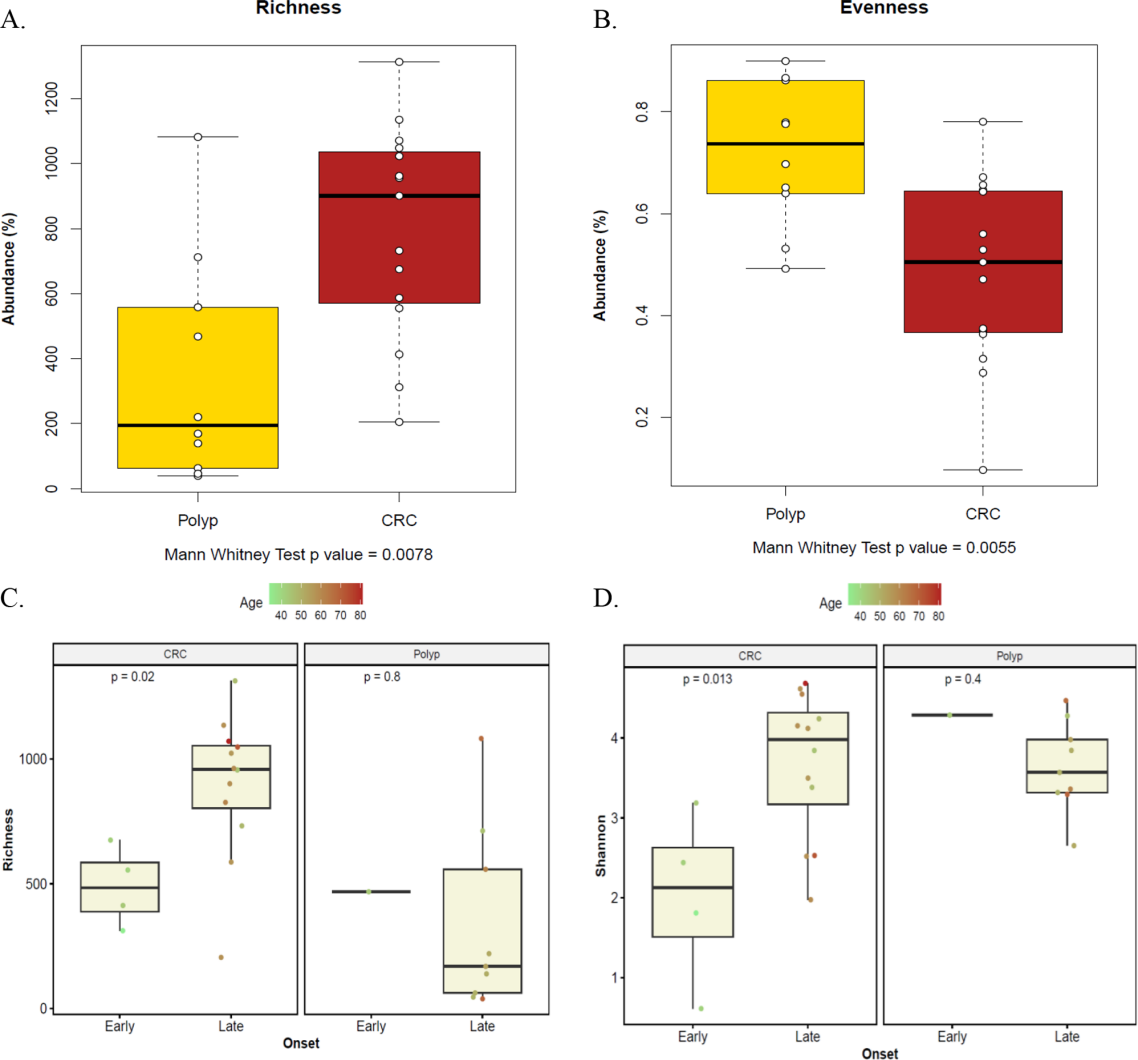
Alpha diversity analysis: species richness index (**A** and **C**), Shannon evenness index (**B**), and Shannon diversity index (**D**) in CRC and colonic polyp groups. Differencess between early and late-onset tumors are specifically shown in panels **C** and **D**. All differences were tested for significance by the non-parametric Mann-Whitney test. *p*-values are shown

Alpha diversity (at the species level) was analyzed and compared between different samples. Overall, species richness within the CRC group was significantly higher than that of the colonic polyps group (Mann Whitney *p-*value = 0.0078, Fig. 4A), while evenness of CRC group was significantly lower than that of the colonic polyps group (Mann Whitney *p*-value = 0.0055, Fig. 4B). Moreover, both species richness and Shannon diversity index of the late onset CRC samples were significantly higher than that of early onset ones (Mann-Whitney *p*-values = 0.02 and 0.013 respectively, Fig. 4C-D).

### Firmicutes/Bacteroidetes (F/B) ratio

**Fig. 5:**
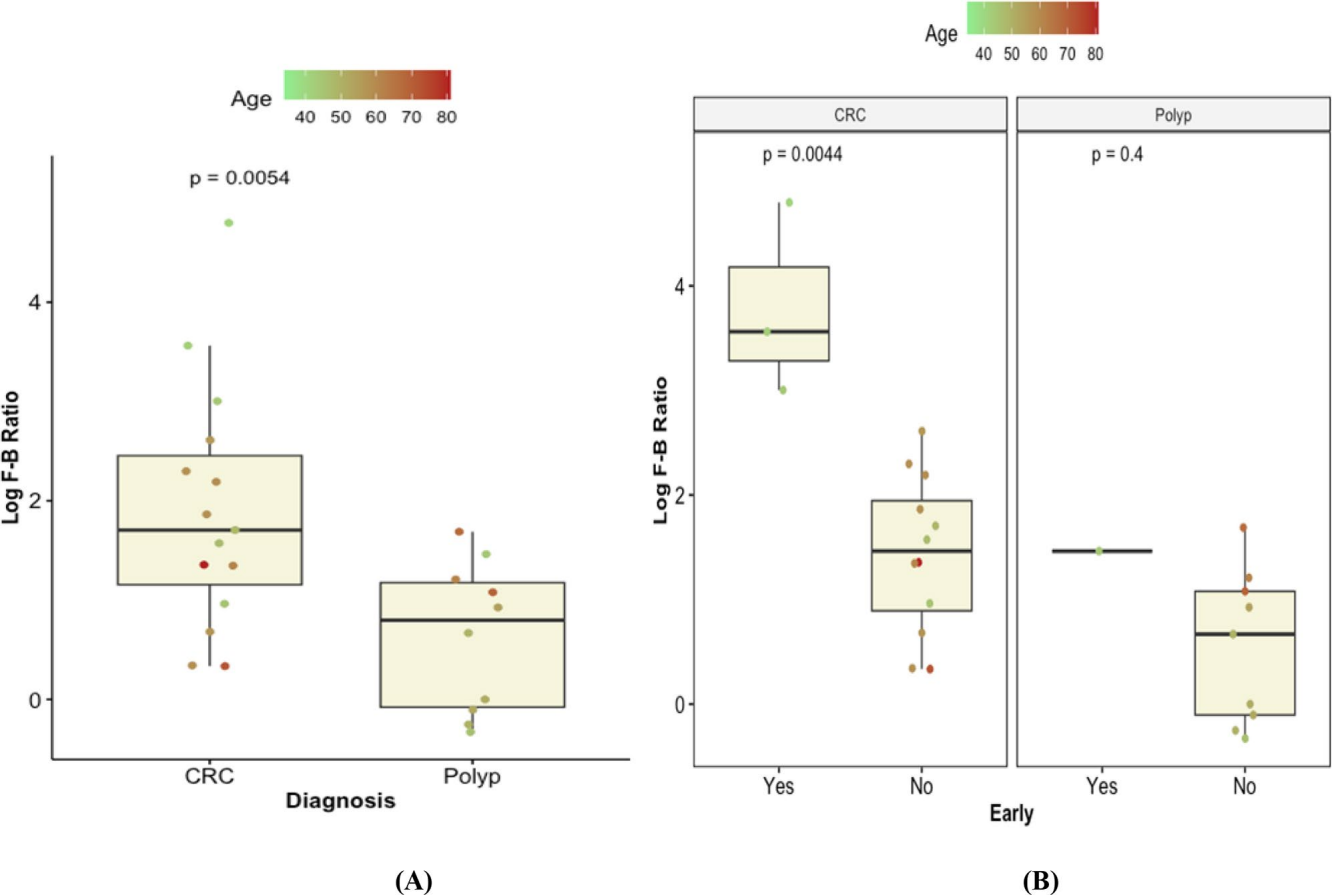
Firmicutes/Bacteroidetes(F/B) ratio (**A**) between CRC and colonic polyps groups, and (**B**) between the early and late onset of CRC and colonic polyps. *p*-values were calculated by Mann-Whitney test. (Yes) refers to early onset, and (No) refers to late onset, while the patient age is used as color gradient for each sample point

The Firmicutes-to-Bacteroidetes (F/B) ratio (or Bacillota-to-Bacteroidota, by the current nomenclature) was among the earlier microbiome biomarkers to be considered as it correlates with several health conditions. In this study, the F/B ratio was significantly higher in the CRC group than in the colonic polyps group *(p*-value = 0.0054). The F/B ratio was also significantly higher in early-onset CRC patients than in late-onset CRC patients (Fig. 5).

### Anatomical site and the age of diagnosis vs. microbial taxon composition

**Fig. 6:**
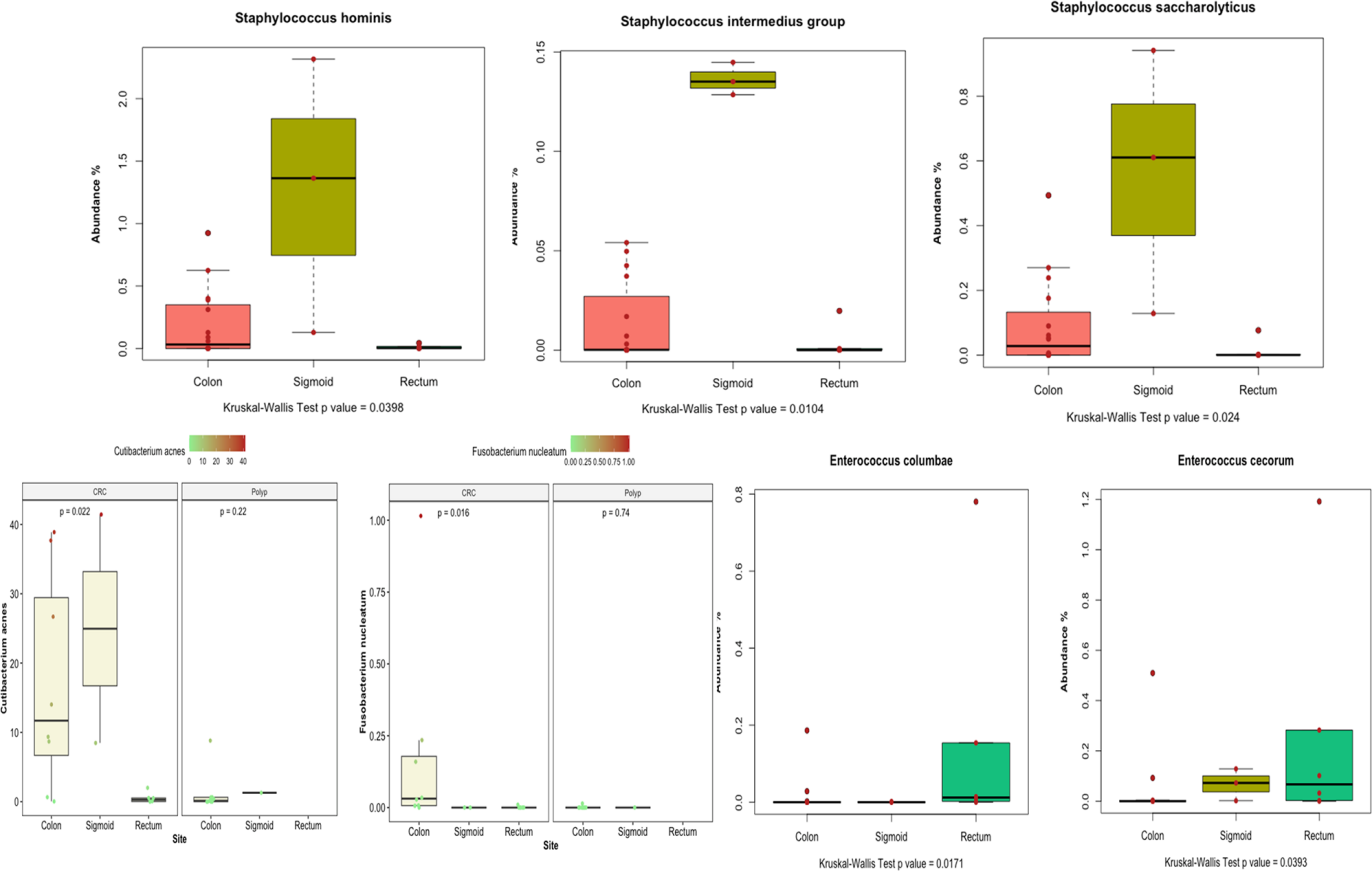
Taxon abundance in CRC and colonic polyps groups split by anatomical site

We also investigated the association between the relative taxon abundance and the anatomical site. The relative abundance of *Staphylococcus* spp. and *Cutibacterium acnes* were significantly higher in samples from sigmoid cancer, while *Enterococcus cecorum* and *Enterococcus columbae* were significantly overabundant in samples with rectum-cancer. The relative abundance of *Fusobacterium nucleatum* was significantly higher in samples from colon cancer (Fig. 6).

**Fig. 7:**
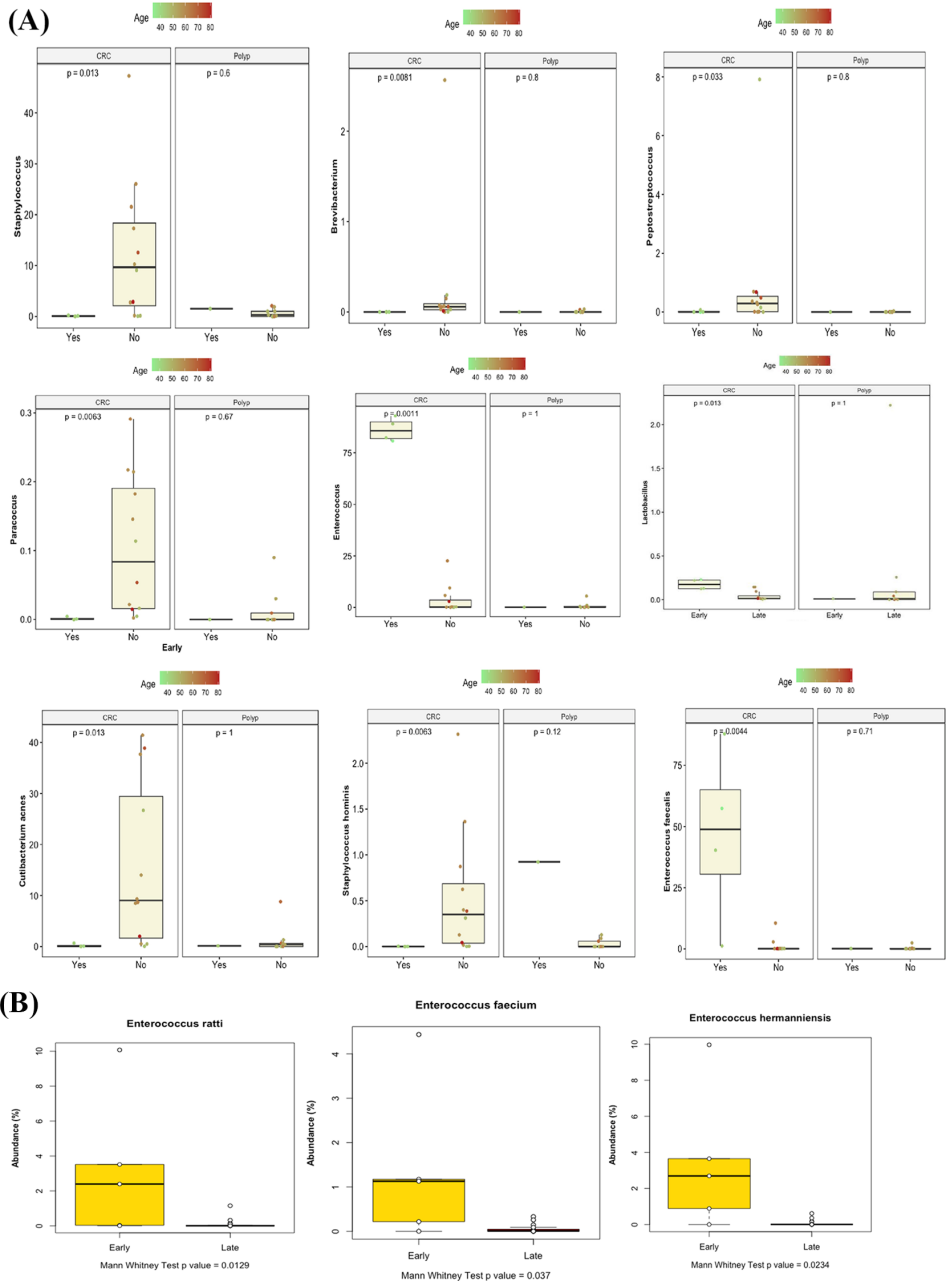
(**A**) Taxon abundance at the genus level in CRC and colonic polyps groups split by age; (**B**) Taxon abundance at the species level in CRC and colonic polyps groups split by age: *p*-values were calculated by Mann-Whitney test; (Yes) refers to early onset, and (No) refers to late-onset

Finally, we investigated the possible differential abundance of some microbial taxa between early (≤ 45 years) and late (> 45 years) onset of CRC and colonic polyps. At the genus level, *Staphylococcus,Peptostreptococcus*, and *Brevibacterium* were significantly more abundant in samples from patients with late onset of CRC, while *Enterococcus* and *Lactobacillus* were significantly more abundant in samples from patients with early onset of CRC (Fig. 7A). At the species level, *Cutibacterium acnes* and *Staphylococcus hominis* were significantly more abundant in samples from patients with late onset of CRC, while *Enterococcus cecorum, Enterococcus columbae, Enterococcus faecalis*, and *Enterococcus faecium* were significantly associated with early onset of CRC (Fig. 7B).

## Discussion

CRC is linked to changes in microbial composition, often known as dysbiosis [20, 21]. Different lifestyle-related factors, such as diet and body weight, may alter the gut microbiota and influence the risk of developing CRC [22]. Genetic and epigenetic alterations brought on by genotoxic stress to the gut microbiota or metabolites in the intestinal environment may result in cancer [23], and the development of CRC may be influenced by the overabundance of particular strains [24]. Most of the findings and associations about the microbiome involvement in CRC, however, are based on studies on fecal samples, which may represent the microbial diversity in the colon, but do dilute the actual composition at the cancerous or adenomatous tissue.

Although a number of excellent studies have identified polyp *vs.* CRC tissue microbiotas, the vast majority—to the best of our knowledge—relied on short-read sequencing technologies. Thus, it offers a broad picture of microbial composition, but—whether it relies on V3-V4 hypervariable region or other variable regions of the 16S rRNA gene lacks sufficient sequence length to resolve many bacterial species. We believe that our approach of using full-length 16S rRNA gene sequencing strengthens some of the prior findings by providing a long-read-based analysis, and adds higher taxonomic resolution at the species level. For example, Hua et al. used Illumina sequencing of the V3–V4 variable region of the 16S rRNA gene to characterize the microbiota differences along the adenoma-carcinoma sequence [25]. In addition, Zhong et al. performed 16S rRNA gene sequencing in normal colorectal mucosa and tissue of colorectal polyps as well as in feces. Their work revealed that *Fusobacterium* and *Streptococcus* were lower in feces both in patients with colorectal polyp and healthy individuals, when compared to those in the normal mucosa in the two groups or in polyp tissues. However, their study did not include CRC tissue samples [26].

Long-read sequencing has just started to be implemented in profiling the microbiota of different body sites or tissues. A recent study conducted Illumina MiSeq sequencing of the V4 region of the 16S rRNA gene to analyze mucosal biopsies collected from multiple colon sites, including healthy controls. This study reported significant alpha diversity differences between CRC and controls but found no clear separation between CRC and polyps. Further characterization of the *Fusobacterium* species and subspecies was performed by MinION nanopore sequencing, confirming their enrichment in CRC, which also agrees with our findings [27]. Another study by Wei et al. used long-read sequencing to classify the fecal microbiota changes associated with colonic adenomatous polyps. Their work provided a broader comparison, including healthy controls, occult blood patients, and adenomatous polyp cases. However, their study did not include CRC tissue samples [28].

The current study used long-read amplicon sequencing to identify bacterial clades at multiple taxon levels (up to the species level) from the tissue microbiome of CRC and colonic polyps, with some initial insights on early- vs. late-onset disease. Our results showed a significant variation in bacterial abundance between the two groups and the age subgroups. The major bacterial phyla detected in this study were Firmicutes, Pseudomonadota, Actinomycetota, Bacteroidetes, and Mycoplasmatota in both the colonic polyps and CRC group, consistent with Russo et al. [29].

It is to be noted that shotgun metagenomics methodologies are often superior to 16S amplicon-based ones as they offer insight into the differential abundance of genes, pathways, and subsystems. However, shotgun metagenomic sequencing is more suitable to stool samples than to tissue samples, as DNA extracted the latter will mostly represent the human host/tumor DNA rather than microbial DNA. Thus, we believe that the choice of long-read nanopore sequencing was the most appropriate for our goal of accurately identifying taxonomic differences between cancerous and adenomatous tissues.

We found that the relative abundance of Firmicutes and Actinomycetota was significantly higher in the CRC group compared with the colonic polyps group. On the other hand, the relative abundance of Pseudomonadota, Bacteroidetes, and Mycoplasmatota was substantially higher in the colonic polyps than in the CRC lesions. A published study reported the abundance of Bacteroidetes in colon cancer, contrary to our findings about its higher relative abundance in patients with colonic polyps; however, that study—like many others—relied on stool analysis and not tumor tissue [30]. As mentioned above, stool samples, while used as proxy for the gut microbiome, are not optimal in cases of localized tumors, as the same intestine will have distinct microbiome signatures at different sites, as confirmed in this work (Fig. 6).

Our data showed that the Firmicutes-to-Bacteroidetes (F/B) ratio was significantly higher in the CRC lesions than in the colonic polyps; this finding is in agreement with previous reports on the higher abundance of bacteria belonging to the Firmicutes phylum in CRC tumors [31]. In line with our findings, Quaglio et al. reported that patients with CRC have shown enrichment with Firmicutes and Bacteroidetes [32]. Of note, a study on Egyptian patients identified a significant reduction in “beneficial Firmicutes” in ulcerative colitis, colorectal adenoma, and CRC when compared to controls [19]. However, this study followed a targeted approach (real-time PCR on 16S rRNA genes of the phylum), which is unable to provide high-resolution taxonomic analysis.

Other studies from Egypt are all based on fecal microbiome profiling: A study by Elkholy et al. examined microbiome dysbiosis in patients with CRC from different ethnic groups, including Egyptians. It analyzed microbiome composition in CRC and normal tissue using short-read 16S rRNA sequencing. Distinct microbial signatures of Egyptian patients were reported compared to African American and European American patients. High abundance of *Herbaspirillum* and *Staphylococcus* was reported in tumor tissues from Egyptian patients [16]. Additionally, an Egyptian pilot study used metagenomic sequencing and investigated gut microbiota in patients with CRC post-colectomy [17]. Another Egyptian study focused on ulcerative colitis patients, highlighting significant gut microbiome dysbiosis. That study demonstrated reduced anti-inflammatory bacteria in ulcerative colitis patients, such as *Firmicutes* and *Faecalibacterium prausnitzii* [18].

In the current work, we found that the bacterial families *Enterococcaceae, Enterobacteriaceae, Propionibacteriaceae*, and *Staphylococcaceae* were relatively more abundant in the CRC group, while *Bacteroidaceae, Morganellaceae, Lachnospiraceae, Yersiniaceae*, and *Erwiniaceae* were more abundant in colonic polyps. At the genus level, the most predominant bacterial genera with high OTUs in the CRC group were *Enterococcus, Cutibacterium, Staphylococcus, Corynebacterium*, and *Peptostreptococcus.* Other bacterial genera were also present in the CRC group but with lower relative abundance, e.g., *Dermabacter, Fusobacterium, Gulosibacter, Parvimonas, Proteus, Prevotella, Bacteroides*, and *Clostridium*. In addition, we found that *Proteus, Prevotella, Bacteroides,Macrococcus, Morganella, Mycolicibacter, Clostridium*, and *Lactobacillus* were significantly more abundant in the colonic polyps than in the CRC lesions.

A major finding here is that CRC lesions had a significantly higher relative abundance of the *Enterococcus* genus when compared with colonic polyps. In line with our findings, Wu et al. used 16S rRNA gene sequencing in previous research and demonstrated that the *Enterococcus* genus was relatively more abundant in patients with CRC than in the healthy controls [33]. In addition, Elahi et al., using TaqMan qPCR, also reported that *Enterococcus* was statistically significantly more abundant in CRC tissue samples [34]; however, TaqMan technology has lower resolution given its targeted nature; thus, confirmation of this finding by our long-read nanopore approach strengthens the results. Other studies agree with ours, by reporting a higher abundance of *Enterococcus* in stool samples from CRC patients than those from healthy controls [35, 36].

*Enterococcus faecalis* is thought to be a driver bacterium in CRC development through inducing inflammation and facilitating epigenetic changes and mutation accumulation [37]. We identified an elevated abundance of *Enterococcus faecalis* in the CRC group than in the colonic polyps group, which agrees with another study reported increased levels of *Enterococcus faecalis* in CRC patients [35]. *Enterococcus faecalis* was also reported to be associated with the onset and progression of CRC [31]. Our findings also propose its possible association with the early onset of CRC, although the data will need to be confirmed by multiple other studies. Previous studies reported that DNA-damaging superoxide radicals and genotoxins produced by *Enterococcus faecalis* may contribute to the CRC development [38, 39].

We also identified *Staphylococcus auricularis* as a prevalent bacterium in the CRC group. This bacterium was previously identified as one of the most common bacteria in healthy external auditory canal (EAC) culture [40], but it is not unusual the find of intraindividual divergence in microbiomes across the human body [41]. In addition, *Gulosibacter hominis* was identified in this study to be more abundant in CRC patients than in patients with colonic polyps. *Gulosibacter hominis* was earlier described as a unique source of opportunistic infections, the most common infections in persons with immunodeficiency [42]. Thus, we might postulate the relationship between this bacterium and a weakened immune system in CRC patients and disease development.

Our findings are in concordance with previously published data, by Osman et al., who reported an over-representation of *Peptostreptococcus stomatis*,* Fusobacterium nucleatum, Parvimonas micra*, and  *Akkermansia muciniphila* in CRC patients when compared with non-CRC controls [43]. We noted the presence of the four formerly mentioned bacterial species in CRC lesions, which had higher relative abundance than in the colonic polyps. The CRC risk estimation analysis conducted using regional differences between Japan, China, the United States, Germany, France, and Austria revealed that *Peptostreptococcus stomatis* is a globally prevalent high-risk pathogen of CRC, and it is a significant variable in CRC risk prediction models worldwide [44]. Here, *Peptostreptococcus stomatis* was found to be much more prevalent in the CRC group. This finding agrees with previously published data from around the world, suggesting a potential role in CRC initiation [43–45]. Moreover, similar results were reported regarding the high abundance of *Peptostreptococcus stomatis* in CRC patients [46].

Strong clinical evidence suggests the association between *Fusobacterium nucleatum* and CRC [47]. It is well documented that *Fusobacterium* spp. are over-represented in CRC tumors, mainly *Fusobacterium nucleatum*, which was previously reported to have a critical role in CRC development [48, 49]. The gut microbiome of CRC patients differed from that of healthy controls, according to a recent study by Arafat et al., who used short-read 16S RNA sequencing to compare microbial diversity in mucosal samples of Kenyan CRC patients to that of healthy controls. Their analysis revealed that *Fusobacterium nucleatum* was present in high concentrations in all CRC patients compared with healthy individuals [50].

Another study matched with our findings identified *Fusobacterium* as CRC-enriched genera [51]. *Fusobacterium nucleatum* has been shown to enhance glycolysis and promote oncogenesis in CRC by up regulating the expression of the lncRNA ENO1-IT1 [52]. It has been emerged also as a critical candidate for CRC predisposition due to its ability to bind to E-cadherin on the surface of colon cells via FadA adhesion, activating the Wnt/B-catenin signaling pathway and producing an inflammatory and oncogenic response, as well as its capacity to bind to the inhibitory immune receptor via Fap2 adhesin, altering natural killer cells [53]. The present study’s findings agree with a large-scale meta-analysis from four cohorts of different ethnicities, using fecal samples’ shotgun metagenomic sequencing, demonstrating abundance of *Parvimonas micra* in CRC patients over healthy controls [54]. Our findings also agree with previous study by Yu et al. that revealed significant higher abundance of *Fusobacterium nucleatum* and *Parvimonas micra* in feces of CRC patients compared to healthy controls [55].

Certain bacteria have shown a protective role against intestinal inflammation, such as *Bacteroides fragilis* [56, 57]. It was reported that polysaccharide A, the immunomodulatory molecule produced by *Bacteroides fragilis*, can induce an anti-inflammatory immune response to prevent intestinal inflammatory diseases in animals with colitis [58]. We found the relative abundance of *Bacteroides fragilis* to be lower in the CRC group, aligning with other studies [59, 60]. We also identified bacterial genera known to be protective against CRC, like *Clostridium* and *Lactobacillus.* Guo et al. reported that most *Clostridium* species have a possible beneficial role in preventing CRC by producing substances such as butyrate [61, 62]. For instance, the probiotic strains of *Lactobacillus* and *Bifidobacterium* were found to be at lower levels in patients with colorectal carcinoma. The protective role was suggested through their ability to secrete antibacterial peptides, compete for adhesion sites, and displace enteropathogens [63]. In addition, other studies revealed that *Lactobacillus* reduces gut inflammation. Such studies reported a significant reduction in the level of *Lactobacillus* in patient groups (polyps and CRC patients) compared with healthy controls [64, 65]. Despite the high translational potential of identifying CRC-protective bacterial species in treating and preventing CRC, research on it is still limited.

## Conclusion

Our results revealed a considerable difference in the overall microbial diversity and the relative abundace of different bacterial taxa between colonic polyps and CRC lesions. Phylum Firmicutes and Actinomycetota were significantly abundant in the CRC group, while phylum Pseudomonadota and Bacteroidota were abundant in the colonic polyps group. The bacterial species *Enterococcus faecalis, Cutibacterium acnes, Peptostreptococcus stomatis, Fusobacterium nucleatum* were significantly enriched in the CRC group, while *Bacteroides fragilis, Proteus mirabilis*, and  *Prevotella corporis* were more abundant in the colonic polyps group. Collectively, we demonstrated the microbial dysbiosis associated with CRC and colonic polyps groups. These findings provide a higher-resolution and more complete microbial profile of the cancerous and noncancerous tissue, which will lead to a better understanding of the pathogenesis of CRC, in general, and in Egyptian patients, in particular.

In addition, we provided initial clinical insights through identifying the microbiota associated with early- and late-onset CRC, as well as anatomical tumor site. The use of long-read nanopore sequencing offers a methodological improvement over previous studies. Future studies will investigate the metabolome profiles of these tissues and lesions to understand the impact of microbiome variations on cellular pathways. In addition, investigating the host-microbiome interaction, in animal models, is crucial to understand the causality and interaction between microbiome and colonic epithelium. Finally, exploring the use of prebiotics and probiotics as adjunctive CRC treatments is also being investigated by several research groups.

### Limitations

Although this study supports our understanding of the tissue microbiome associated with CRC and colonic polyps in Egyptian patients, a larger sample size would provide a higher resolution and an ability to resolve subgroups based on tumor type, stage, as well as interindividual differences. Multinational studies will enable a more comprehensive determination of the microbiota contributing to CRC development.

## Electronic supplementary material

Below is the link to the electronic supplementary material.

Supplementary Material 1

Supplementary Material 2

Supplementary Material 3

## Data Availability

All data generated or analyzed during this study and its supplementary information files are included in this article and any raw data can be obtained from the corresponding authors upon request.
