# Supplementary material for: Long-read 16S rRNA amplicon sequencing reveals microbial characteristics in patients with colorectal adenomas and carcinoma lesions in Egypt
Source: Gut Pathog. 2025 Feb 2;17:8. doi: 10.1186/s13099-025-00681-9 (PMC11789410; doi:10.1186/s13099-025-00681-9)
Supplement: Supplementary file 1 — Supplementary Material 1 [file 13099_2025_681_MOESM1_ESM.docx]

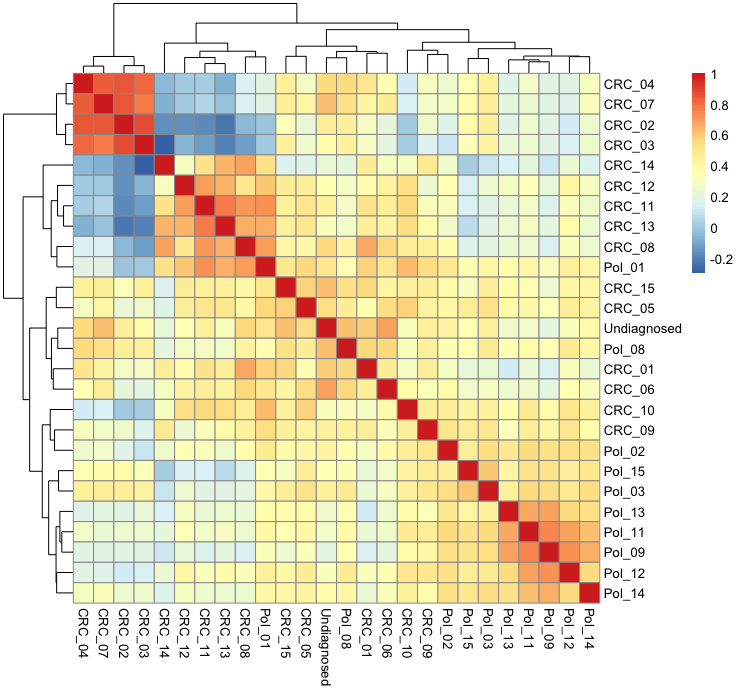


**Supplementary figure 1: Heat map represents the five clusters of correlated relative abundance within the microbiome profiles of samples from CRC tissue and colonic polyps**
