# Supplementary material for: Long-read 16S rRNA amplicon sequencing reveals microbial characteristics in patients with colorectal adenomas and carcinoma lesions in Egypt
Source: Gut Pathog. 2025 Feb 2;17:8. doi: 10.1186/s13099-025-00681-9 (PMC11789410; doi:10.1186/s13099-025-00681-9)
Supplement: Supplementary file 3 — Supplementary Material 3 [file 13099_2025_681_MOESM3_ESM.docx]

**Supplementary Table 2: The significantly abundant bacterial phyla and families in CRC and colonic polyps**

| **Taxon level** | **Taxa Name** | **Tax ID** | **Relative Abundance in CRC group (%)** | **Relative Abundance in Polyps group (%)** | ***p*-value** |
| --- | --- | --- | --- | --- | --- |
| **Phylum** | Bacillota (Firmicutes) | 1239 | 46.09 | 22.09 | 4.600075e-02 |
|  | Pseudomonadota | 1224 | 25.53 | 42.96 | 4.165436e-02 |
|  | Actinomycetota | 201174 | 24.24 | 7.20 | 3.739349e-02 |
|  | Bacteroidota | 976 | 2.79 | 11.40 | 1.274379e-02 |
|  | Mycoplasmatota | 544448 | 0.02 | 6.00 | 7.733711e-03 |
|  | Cyanobacteriota | 1117 | 0.15 | 1.38 | 1.774770e-02 |
|  | Log Firmicutes/ Bacteroidota (F/B) ratio | - | 1.78 | 0.79 | 5.474770e-02 |
| **Family** | *Enterococcaceae* | 81852 | 24.59 | 3.46 | 5.601075e-03 |
|  | *Enterobacteriaceae* | 543 | 16.16 | 10.67 | 3.165336e-02 |
|  | *Propionibacteriaceae* | 31957 | 17.46 | 3.65 | 2.739349e-03 |
|  | *Staphylococcaceae* | 90964 | 8.91 | 3.18 | 2.273379e-03 |
|  | *Peptostreptococcaceae* | 186804 | 1.83 | 0.22 | 6.733411e-03 |
|  | *Dermabacteraceae* | 85020 | 1.73 | 0.10 | 3.744770e-02 |
|  | *Streptococcaceae* | 1300 | 1.18 | 0.57 | 6.474750e-02 |
|  | *Fusobacteriaceae* | 203492 | 0.51 | 0.17 | 1.920015e-02 |
|  | *Brevibacteriaceae* | 85019 | 0.33 | 0.05 | 2.218529e-02 |
|  | *Bacteroidaceae* | 815 | 1.81 | 9.45 | 2.587850e-03 |
|  | *Morganellaceae* | 1903414 | 0.55 | 6.67 | 5.601075e-03 |
|  | *Lachnospiraceae* | 186803 | 2.28 | 6.50 | 4.165336e-02 |
|  | *Oscillospiraceae* | 216572 | 1.16 | 4.82 | 2.739349e-02 |
|  | *Prevotellaceae* | 171552 | 0.75 | 4.46 | 2.273379e-03 |
|  | *Yersiniaceae* | 1903411 | 2.39 | 5.21 | 1.285308e-01 |
|  | *Erwiniaceae* | 1903409 | 1.63 | 5.14 | 1.982071e-01 |
|  | *Bacillaceae* | 186817 | 1.26 | 2.06 | 3.375926e-02 |
|  | *Lactobacillaceae* | 33958 | 0.36 | 1.14 | 1.376175e-02 |

**Supplementary Table 3: Abundant bacterial genera and species among CRC and colonic polyps**

| **Taxon level** | **Taxa Name** | **Tax ID** | **Abundance in CRC group (%)** | **Abundance in Polyps group (%)** | ***p*-value** |
| --- | --- | --- | --- | --- | --- |
| **Genus** | *Enterococcus* | 1350 | 24.28 | 3.36 | 8.614224e-04 |
| **Species** | *Enterococcus faecalis* | 1351 | 12.67 | 2.22 | 3.895108e-02 |
|  | *Enterococcus ratti* | 150033 | 1.36 | 0.20 | 1.342268e-02 |
|  | *Enterococcus hermanniensis* | 249189 | 1.30 | 0.21 | 1.053951e-02 |
|  | *Enterococcus canis* | 214095 | 1.14 | 0.15 | 1.190069e-02 |
|  | *Enterococcus casseliflavus* | 37734 | 0.92 | 0.17 | 1.769732e-02 |
|  | *Enterococcus asini* | 57732 | 0.85 | 0.18 | 1.284308e-02 |
|  | *Enterococcus durans* | 53345 | 0.65 | 0.49 | 1.920015e-02 |
|  | *Enterococcus faecium* | 1352 | 0.54 | 0.12 | 2.218529e-02 |
|  | *Enterococcus malodoratus* | 71451 | 0.43 | 0.08 | 1.587850e-02 |
|  | *Enterococcus saccharolyticus* | 41997 | 0.39 | 0.10 | 8.297254e-03 |
|  | *Enterococcus sulfurous* | 1356 | 0.29 | 0.06 | 9.967594e-03 |
|  | *Enterococcus mundtii* | 53346 | 0.27 | 0.05 | 1.577368e-02 |
|  | *Enterococcus cecorum* | 44008 | 0.19 | 0.10 | 1.284308e-02 |
| **Genus** | *Cutibacterium* | 1912216 | 17.01 | 3.60 | 1.983071e-02 |
| **Species** | *Cutibacterium acnes* | 1747 | 12.02 | 2.51 | 3.377926e-02 |
| **Genus** | *Staphylococcus* | 1279 | 8.60 | 2.95 | 1.379175e-03 |
| **Species** | *Staphylococcus epidermidis* | 1282 | 0.50 | 0.30 | 1.342268e-02 |
|  | *Staphylococcus hominis* | 1290 | 0.43 | 0.41 | 4.512807e-02 |
|  | *Staphylococcus auricularis* | 29379 | 0.26 | 0.18 | 1.322031e-02 |
|  | *Staphylococcus warneri* | 1292 | 0.24 | 0.17 | 2.401883e-03 |
| **Genus** | *Corynebacterium* | 1716 | 2.86 | 0.79 | 9.837196e-03 |
| **Species** | *Corynebacterium jeikeium* | 38289 | 0.91 | 0.02 | 2.154708e-02 |
|  | *Corynebacterium minutissimum* | 38301 | 0.55 | 0.07 | 4.752006e-02 |
| **Genus** | *Peptostreptococcus* | 1257 | 0.91 | 0.01 | 4.903057e-04 |
| **Species** | *Peptostreptococcus stomatis* | 341694 | 0.64 | 0.00 | 9.493397e-05 |
| **Genus** | *Dermabacter* | 36739 | 1.41 | 0.04 | 7.464618e-03 |
| **Species** | *Dermabacter vaginalis* | 1630135 | 1.03 | 0.03 | 7.464618e-03 |
| **Genus** | *Fusobacterium* | 848 | 0.49 | 0.16 | 1.247135e-02 |
| **Species** | *Fusobacterium nucleatum* | 851 | 0.21 | 0.02 | 3.803178e-02 |
| **Genus** | *Gulosibacter* | 256818 | 0.39 | 0.09 | 4.817166e-02 |
| **Species** | *Gulosibacter hominis* | 2770504 | 0.44 | 0.02 | 4.223712e-02 |
| **Genus** | *Akkermansia* | 239934 | 0.38 | 0.05 | 4.223712e-02 |
| **Species** | *Akkermansia muciniphila* | 239935 | 0.36 | 0.05 | 4.223712e-02 |
| **Genus** | *Brevibacterium* | 1696 | 0.32 | 0.04 | 1.910627e-02 |
| **Species** | *Brevibacterium ravenspurgense* | 479117 | 0.33 | 0.02 | 2.927237e-02 |
| **Genus** | *Oligella* | 90243 | 0.31 | 0.00 | 4.354502e-02 |
| **Species** | *Oligella urethralis* | 90245 | 0.31 | 0.00 | 4.354502e-02 |
| **Genus** | *Parvimonas* | 543311 | 0.27 | 0.07 | 3.877457e-03 |
| **Species** | *Parvimonas micra* | 33033 | 0.24 | 0.07 | 4.461644e-03 |
| **Genus** | *Proteus* | 583 | 0.06 | 4.31 | 5.144341e-04 |
| **Species** | *Proteus mirabilis* | 584 | 0.03 | 2.73 | 1.051433e-02 |
| **Genus** | *Prevotella* | 838 | 0.29 | 3.80 | 2.267416e-04 |
| **Species** | *Prevotella corporis* | 28128 | 0.01 | 0.32 | 2.503537e-02 |
| **Genus** | *Bacteroides* | 816 | 1.13 | 2.20 | 4.354502e-02 |
| **Species** | *Bacteroides fragilis* | 817 | 0.46 | 1.15 | 4.889423e-02 |
| **Genus** | *Macrococcus* | 69965 | 0.02 | 1.52 | 4.817166e-02 |
| **Genus** | *Morganella* | 581 | 0.15 | 1.43 | 3.858861e-03 |
| **Genus** | *Mycolicibacter* | 1073531 | 0.01 | 1.36 | 9.837196e-03 |
| **Genus** | *Clostridium* | 1485 | 0.33 | 0.87 | 2.503537e-02 |
| **Genus** | *Lactobacillus* | 1578 | 0.07 | 0.34 | 2.078367e-03 |
